# Breast cancer research and treatment reconstruction of unilateral breast structure using three-dimensional ultrasound imaging to assess breast neoplasm

**DOI:** 10.1007/s10549-019-05202-2

**Published:** 2019-04-05

**Authors:** Yuanyuan Lu, Junlai Li, Xiaohui Zhao, Jie Li, Jie Feng, Erlong Fan

**Affiliations:** 1 Department of Ultrasound, Chinese People’s Liberation Army General Hospital, The Southern Building, 28 Fuxing Road, Beijing, 100853 China; 2 Department of General Surgery, Chinese People’s Liberation Army General Hospital, 28 Fuxing Road, Beijing, 100853 China; 3 Department of Radiology, Chinese People’s Liberation Army General Hospital, 28 Fuxing Road, Beijing, 100853 China

**Keywords:** Breast neoplasm, Tumor size, Ultrasound (US), Magnetic resonance imaging (MRI)

## Abstract

**Purpose:**

To develop and evaluate the accuracy of a three-dimensional (3D) US method for assessing unilateral breast reconstruction and discuss the feasibility of breast ultrasound 3D reconstruction of the unilateral breast compared with 3D MRI.

**Methods:**

Sixty-four breast lesions were collected for surgical resection. (1) MRI and US imaging were used to reconstruct the 3D models of the breast neoplasm. The diameters for maximum length, width, and depth of the negative tumor margins were used as the primary standards for comparison. (2) The measurement direction was determined by the largest gravity change between the two body positions. (3) The vertical distance from the midpoint of breast neoplasm to the ipsilateral nipple was calculated via MRI and US reconstruction.

**Results:**

(1) Comparison of the measured size and histopathology of the breast neoplasm showed that US, MRI, and histopathology were highly correlated (*p* < 0.001). (2) When compared with the other two vertical directions, the direction with the largest gravity change had the greatest difference between MRI and US measurements. (3) The vertical distance from the breast neoplasm to the ipsilateral nipple and skin junction was significantly different (*p* > 0.05).

**Conclusions:**

We have presented a novel US 3D reconstruction method for evaluating tumor size, which can provide a basis for investigated advanced visualization techniques for assessing breast tissue such as holographic presentation of 3D image data. These methods can provide physicians with a novel approach for making accurate surgical plans, for better communication with patients, and for more effective navigating throughout the operation.

## Objectives

Ultrasound (US), molybdenum target mammography, and magnetic resonance imaging (MRI) are the major imaging methods for evaluating space-occupying breast lesions. They provide information on the size, morphology, infiltration range, and location of the breast lesions, which is helpful in developing effective treatment plans [1]. On one hand, conventional two-dimensional images combined with textual descriptions only provide limited information to physicians [2]. On the other hand, the current imaging techniques that can achieve a full three-dimensional (3D) reconstruction of the breast structure also have several issues [3, 4]. The patient positioning in MRI and molybdenum target mammography are different from that during surgery; therefore, the position of the breast neoplasm cannot be accurately obtained. Breast CT also exposes the patients to radiation; thus, its use is limited. US is considered an effective modality for determining tumor size and histopathology [5–7], and patient positioning in US is completely consistent with that in surgery. Reconstruction of the complete structure of the breast in 3D via ultrasound (US) can provide comprehensive preoperative information on the size, morphology, and location of the lesion [3, 8, 9], which ultimately facilitates accurate preoperative planning, improves physician–patient communication, and provides new ideas for visualization during intraoperative navigation [10–16]. In this study, we aimed to develop a three-dimensional (3D) US method for reconstruction of unilateral breast structure, which could allow surgeons to obtain 3D images on the size, morphology, and location of breast tumors before surgery.

## Materials and methods

**Fig. 1:**
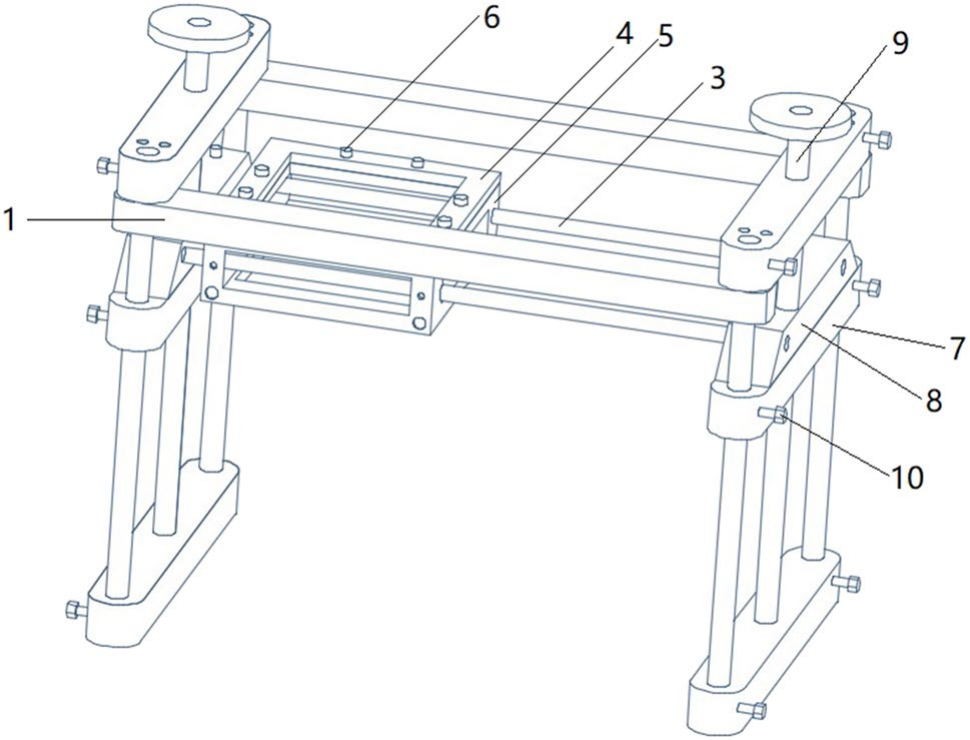
Sink design Positive

**Fig. 2:**
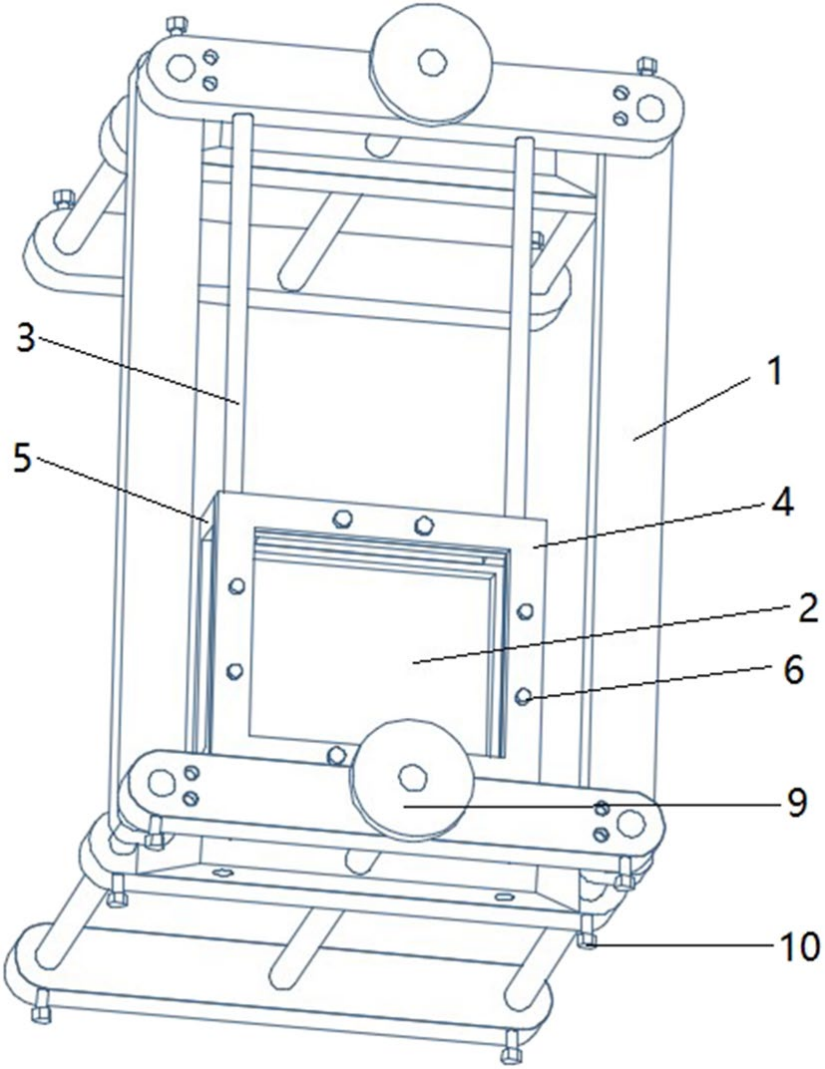
Sink design profile: (1) Frame, (2) Seal groove, (3) Slider, (4) Upper frame, (5) Lower frame, (6) Bolt, (7) Lift adjusting plate, (8) Slider support plate, (9) Adjuster screw, (10) Fixing screw

**Fig. 3:**
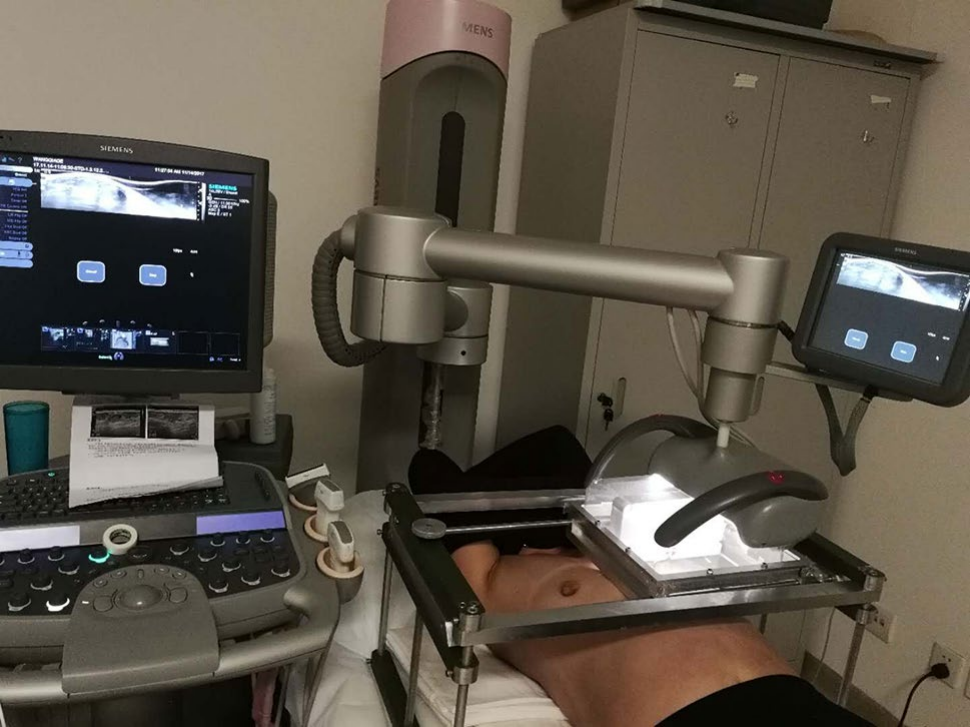
Examination of the patient with ABVS + sink

1. Clinical data: A total of 64 breast lesions (including 15 benign lesions and 49 malignant lesions) were collected from 57 patients who visited the breast surgery department of the People’s Liberation Army (PLA) General Hospital between January 2017 and April 2018. The inclusion criteria were (1) age 18–85 years; (2) confirmed diagnosis of solid space-occupying breast lesions; (3) localized lesions, with no diffuse lesions, and clear boundaries; (4) the breast surgery was planned to be performed in our hospital; and (5) signed consent. A total of 3, 5, and 10 patients who had unclear lesion boundaries, diffuse lesions, and overly large breasts that exceeded the scanning range of the probe, respectively, were excluded. Thus, 39 patients were included in the final analysis. This study was approved by the Ethics Committee of the PLA General Hospital, and all patients provided written informed consent before automated breast volume scanner (ABVS) examination.
2. Instrument: Ultrasonography was performed using Siemens S2000 ABVS. The probe length was 20 cm; scanning depth, 20 cm; slice thickness, 0.525 mm; and the machine was set to continuous automatic tomography. MRI was performed using GE750 (General Electric Company, GE, US) (Siemens Trio; 3.0 T, 8-channel coil, and 1-mm slice thickness). The interval between surgery and each examination was less than 7 days.
3. Research methods: The specific methods for full 3D reconstruction of the unilateral breast structure in patients with space-occupying breast lesions are as follows: The Siemens S2000 ABVS breast probe has a scanning range of 20 × 20 cm, which could be used to measure most unilateral breast sizes in the Chinese population. However, the ultrasonic probe has a flat surface, making it unable to develop full imaging of the convex structure of the breast. In addition, the ABVS probe generates pressure during the examination process, leading to deformation of the breast lesion. To overcome these limitations, we have designed and developed a sealed silicone sink with adjustable stands (Figs. 1,2,3). Water has ultrasonic permeability and deformation property. With the help of water, the unilateral breast of the patient can have a level contact with the probe of the ABVS ultrasonic robot arm, which ensures that the scanning range covers the entire unilateral breast. Concurrently, the breasts were exposed to as little external pressure as possible to reduce the deformation of the lesion. The sealing function of water tank is first confirmed through the silicone film at the bottom of the water tank. The outer layer of the silicone film and the breast surface need to be coated with an appropriate amount of coupling agent to achieve a connection between the silicone film and the breast surface. The coupling agent should be adequate to meet the requirements of complete coverage. However, it is important not to add too much coupling agent as this can influence the quality of the doped gas image.

## Segmentation and 3D reconstruction

**Fig. 4:**
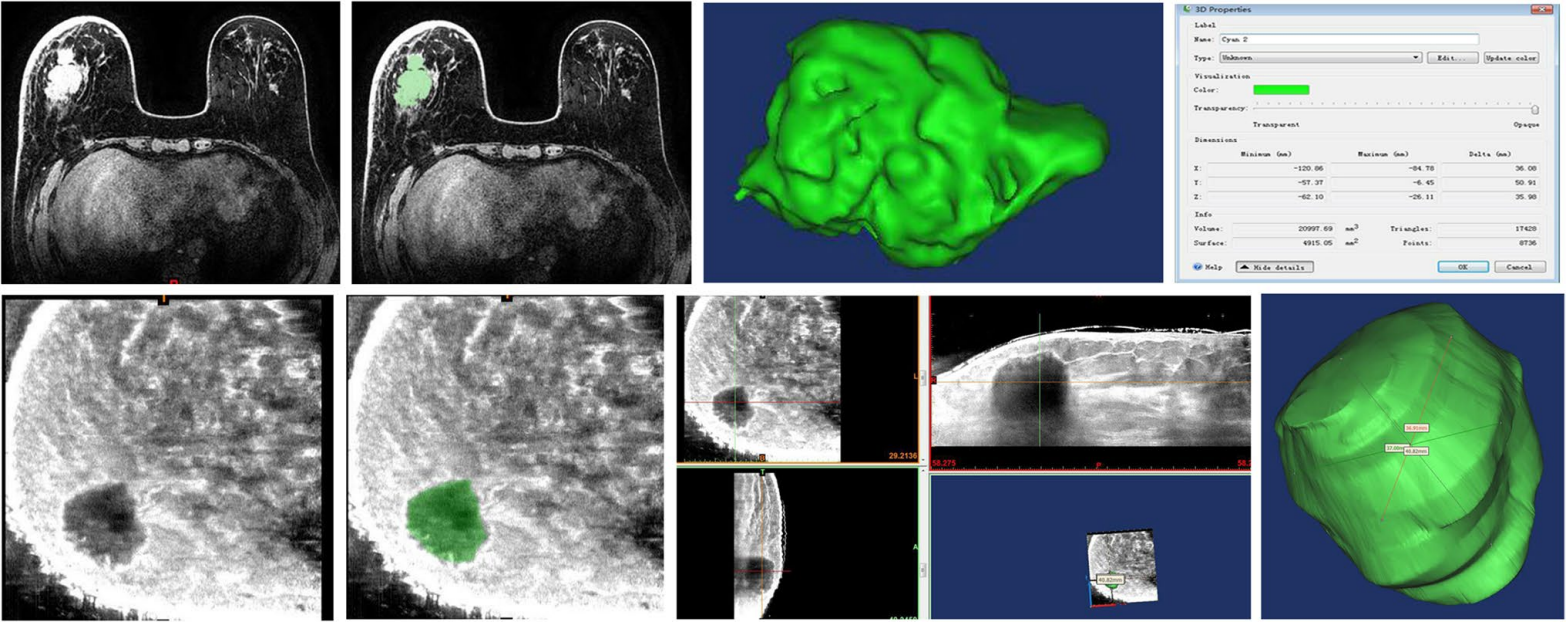
Three-dimensional MRI reconstruction flowchart; three-dimensional ultrasound reconstruction flowchart

The breast image DICOM data obtained via MRI and the ABVS US scanner were imported into Mimics 18.0 software (Materialise). Manual segmentation was performed on the basis of the threshold-based method, as described in Eq. (1) (The threshold represents a parameter for each voxel of data, and the calculation method is obtained by subtracting the difference (delta) from the block range average centered on this pixel. The delta value is constantly adjusted in the experiment, and the surface rendering of the segmentation result was updated in real-time until a better segmentation result was obtained). The 3D model was constructed and saved in STL format once the segmentation was completed. The 3D reconstruction of the *x, y*, and *z* axes of the tumor was generated automatically.

1 $$I(x,y,z)=\left\{ {\begin{array}{{ll}} {1,\quad {\text{if}}\;\;{\text{1}}(x,y,z) \geq {\text{delta}}} \\ {0,\quad {\text{else}}} \end{array}} \right.$$

where *I* (*x, y, z*) is the intensity value of voxel (*x, y, z*), and delta is the threshold value (Fig. 4).

## Observation and evaluation criteria

1. The maximum values of *x, y*, and *z* axes of the tumor obtained by breast ultrasound and breast MRI after tumor segmentation were compared with the tumor pathology (the diameters of maximum length, width depth of the tumor were measured according to the negative tumor margins). A measurement difference range < 5% was considered acceptable.
2. The maximum size of the breast neoplasm was measured in the direction vertical to the ground and in two other directions that are perpendicular to this direction. The diameter differences between the US and MRI reconstructed breast neoplasm were compared, and the effects of gravity on patient positioning were analyzed.
3. The vertical distance from the midpoint of the breast neoplasm to the ipsilateral nipple and skin junction was calculated. The effect of patient positioning change (breast MRI was performed in the prone position, where breast US was performed in the supine position) on the spatial location of the tumor was evaluated.

### Statistical analyses

All data were analyzed using SPSS 19.0 and GraphPad statistical mapping software. The normal distribution of the measured data was represented as *x* ± *s*, and the non-normal distribution was represented as *M*(QR). Pathological measurements were used as a gold standard, and Spearman’s rank correlation coefficient and the Bland–Altman plot were used to evaluate the correlation and consistency in the measurement of tumor size between histopathology-MRI and histopathology-US, respectively. *p* < 0.05 was considered significant.

### Quality control

(1) The above examination methods were all completed by our regular examination physicians. The images were manually segmented and measured by designated radiologists along with 3D reconstruction personnel. (2) The database was managed by a specialist.

## Results

### Patient and tumor characteristics

The average age of the cohort was 44.4 years (range 24–61 years). The nodule sizes ranged from 3 to 73.5 mm. Histopathologically, 15 patients had fibroadenoma (23.4%); 48, invasive ductal carcinoma (75%); and 1, medullary carcinoma (1.6%).

### MRI and US reconstruction

**Fig. 5:**
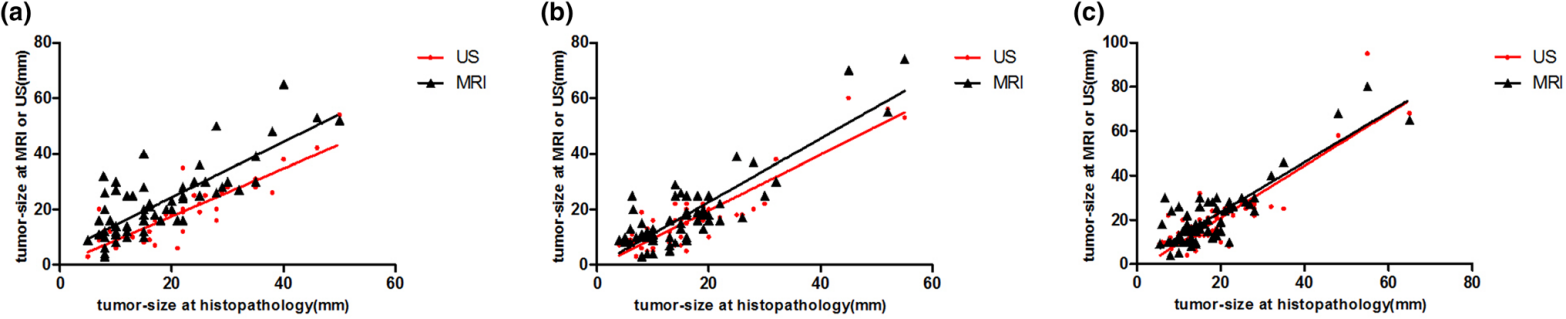
Values of three-dimensional warp knits of breast neoplasm measured via MRI, ultrasound, and histopathology. (The difference measured between MRI and histopathology, the difference measured between US and histopathology, i.e., the correlation between MRI, US, and histopathological measurements, respectively) Three dimensions were used for the correlation test based on the axial direction: **a** Longest diameter (US: *r* = 0.87, *p* < 0.0001, MRI: *r* = 0.86, *p* < 0.0001). **b** Widest diameter (US: *r* = 0.88, *p* < 0.0001, MRI: *r* = 0.79, *p* < 0.0001). **c** Deepest diameter (US: *r* = 0.88, *p* < 0.0001, MRI: *r* = 0.86, *p* < 0.0001). *p* < 0.05 was considered significant

**Fig. 6:**
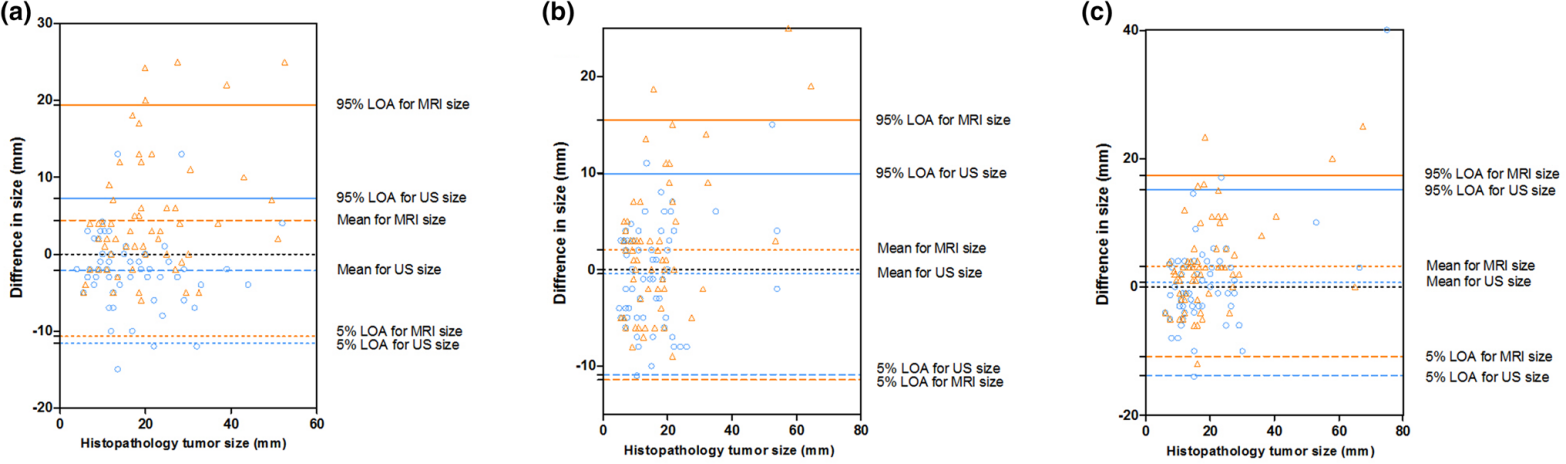
Combination of Bland–Altman plot for the differences between imaging modalities and the histopathological size for comparison. The circle indicates the difference in the mean size between the ultrasound and histopathological measurement. The times indicate the difference in the mean size between mammographic and histopathological measurements. The triangle indicates the difference in the mean size between magnetic resonance imaging and histopathological evaluation. **a** Longest tumor size. **b** Deepest tumor. **c** Widest tumor size

MRI measurements correctly estimated the histopathology in 50% of the cases, overestimated it in approximately 29.7% of the cases, and underestimated it 20.3% of the cases. US measurements correctly estimated the histopathology in 62.5% of the cases, overestimated it in 11% of the cases, and underestimated it in 26.5% of the cases (Table 1). The similarity coefficients of both MRI and US measurements were very close on the *x* and *z* axis, whereas the biggest difference was on the *y* axis. Although both examination methods had high similarity coefficients when compared with histopathology, US showed a higher similarity. *X* axis: US (*r*: 0.87; CI 0.6855–0.8720), MRI (*r*: 0.86; CI 0.7864–0.9161); *y* axis: US (*r*: 0.88; CI 0.8192–0.9297), MRI (*r*: 0.79 CI 0.6855–0.8720); *z* axis: US (*r*: 0.88; CI 0.8187–0.9295), MRI (*r*: 0.86; CI 0.7856–0.9157) (Figs. 5,6).

**Table 1:** US, MRI, and histopathologic measurements (mean ± SD) in each of maximum diameters of different axles and the rate of correctly, overestimated, underestimated venus final pathology (*n* = 64)

	*X* (mm)	*Y* (mm)	*Z* (mm)	US, MRI venus final pathology		
				Correctly	Overestimated	Underestimated
PA	17.83 ± 10.81	17.67 ± 10.19	15.72 ± 10.10			
US	18.51 ± 14.54	15.48 ± 9.89	15.25 ± 11.48	62.5%	11%	26.5%
MRI	21.08 ± 14.07	22.05 ± 12.71	17.75 ± 13.34	50%	29.7%	20.3%

*X* the tumor largest diameter, *Y* the tumor deepest diameter, *Z* the tumor widest diameter (measurement difference range < 5% was considered acceptable)

During the examination, the 3D MRI reconstruction of the breast achieved higher boundary clarity when compared with the US. The image quality was not affected by the size and structure of the breast or by the photoacoustic effect of the breast neoplasm. The repeatability of the 3D MRI reconstruction was very high. However, the 3D US reconstruction of the breast was affected by clarity instability on the boundaries of tumor during manual segmentation.

In particular, it was influenced by the halo sign around the tumor and the echo signal attenuation behind the tumor, resulting in a low repeatability and increased difference in the results. In the present study, contrast-enhanced MRI was performed. The use of the contrast dye allowed for clear visualization of the boundaries and internal structures of the tumors, resulting in a high contrast to the normal tissues. By contrast, no contrast agents were used in US, which resulted in a less clear contrast between the tumor boundaries and the normal tissues. In addition, echo signal attenuation occurred in the vertical direction of the ultrasonic probe, leading to unclear segmentation of the boundaries during reconstruction and affecting accurate evaluation of the measurement result.

**Fig. 7:**
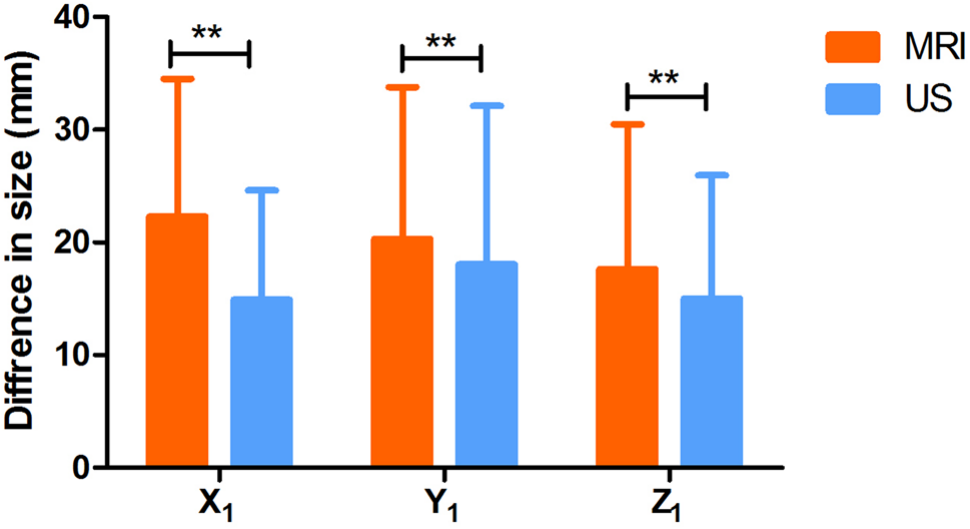
Measurement direction of the breast neoplasm was determined according to the gravity change, and the difference in tumor size between those achieved via three-dimensional magnetic resonance imaging (MRI) and ultrasound (US) reconstruction was determined. x1: maximum mean value in the sagittal position, x2: maximum mean value in the horizontal position, x3: maximum mean value in the coronal position (**p < 0.01)

In the study, we found that the measured value of MRI was a significantly higher estimation than that of US on the *Y* axis. Because of the differences in the examination positions between the two breast examination methods (MRI and US), morphological changes in the space-occupying lesions caused by the breast deformation lead to differences in the measurement results. With MRI, patients are in the prone position, with the breast tumor in the sagittal position, creating a gravitational pull of the breast toward the nipple of the affected side. However, in the ultrasound examination, the sagittal position of the breast tumor has no such effect. Instead, gravity acts in the opposite direction to the nipple (including gravity of the breast tissue and the pressure between the ultrasound probe and the gland). 3D reconstruction of the breast mass lesions was conducted in the sagittal, coronal, and horizontal directions of the body to measure the maximum values in each direction of the tumor, represented by x1, y1, and z1, respectively. Finally, we found that although there was a high correlation coefficient in the sagittal, coronal, and horizontal directions, there was no significant difference. In the sagittal direction, the difference between the two examination methods was found to be the largest (NMR was higher than the ultrasonic value). The horizontal difference perpendicular to it is the smallest (Fig. 7; Table 2), which is similar to the result that we initially expected. It is believed that the gravitational effect caused by postural changes is one of the possible reasons for the high estimation of MRI on the *Y* axis.

**Table 2:** Mean values, standard deviations, and p-values of the measurements obtained via magnetic resonance imaging (MRI) and ultrasound (US) reconstruction in sagittal, horizontal, and coronal positions

	MRI		US		T	P
X	22.27	12.23	14.92	9.694	8.187	< 0.0001
Y	20.31	13.48	18.04	14.06	2.998	0.0039
Z	17.63	12.85	15	10.94	3.512	0.0008

**Fig. 8:**
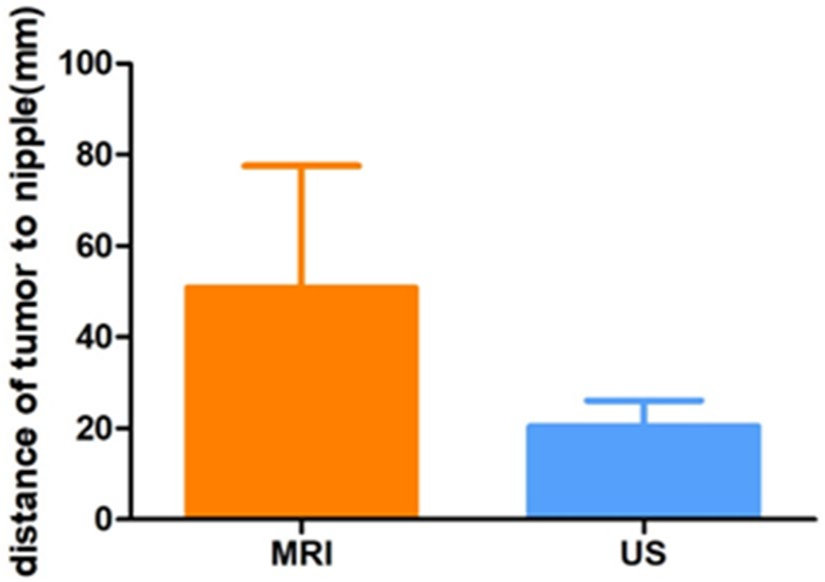
Comparison of the distance of the tumor to the nipple as measured via three-dimensional magnetic resonance imaging (MRI) and ultrasound (US) reconstruction (**p < 0.01)

There were significant differences in the distance from the tip of the tumor to the bottom of the nipple as measured via breast MRI with the patient in the prone position and US reconstruction in the supine position. With the MRI, the maximum distance from the tumor to the nipple was 121.4 mm, the minimum distance was 20.4 mm, and the average was 50.8 mm. However, for the US examination, the maximum distance was 33.8 mm, the minimum was 9.8 mm, and the average was 20.4 mm. The average ratio of the two examinations was greater than 2.5 (Fig. 8; Table 3), indicating that different patient positioning could lead to significantly different measurements in the location of the breast space-occupying lesions.

**Table 3:** The mean and standard deviation (SD) of the tumor–to-nipple distances measured via 3-dimensional magnetic resonance imaging (MRI) and ultrasound (US)

Method	Mean	SD	r	t-Paired
MRI	50.7781	26.72554	0.618**	10.253**
US	20.4375	5.60543		

**Indicates *p* < 0.01

## Discussion

In this study, we found that the MRI and US measurements were significantly different on the *y* axis, and MRI yielded a higher estimation value than US. Patient positioning varied between the two examination methods, and positioning may deform the mammary gland and in turn cause morphological changes of the lesions, resulting in different measured values. In breast MRI, patients are placed in a prone position and there was a pulling effect in the sagittal position of the breast neoplasm toward the ipsilateral nipple due to gravity. This was not an issue in US examination as the patient was positioned supine. Instead, the effect was in the opposite direction of the nipple also caused by gravity (including the gravity of the mammary gland and the pressure between the US probe and the gland).

The maximal values of breast space-occupying lesions were measured in the sagittal, coronal, and horizontal positions of the body after the 3D reconstruction, and these values were represented as x1, y1, and z1. We found that although the correlation coefficient was high in the sagittal, coronal, and horizontal positions, there was no significant difference. The difference between the two examination methods was the greatest in the sagittal position (the measured value of MRI was greater than the US), whereas it was the smallest in the horizontal position. It is possible that the gravity effect caused by the change in patient positioning could lead to the high estimation value on the *y* axis on MRI reconstruction.

We found that the sink design not only enabled us to obtain a full visualization of the breast boundaries but also removed additional pressure from the probe on the mammary gland. In addition, the patient positioning during ultrasonography was very similar to the position in the surgery. However, although the US reconstruction had a high correlation coefficient to the histopathology, the images obtained were not as clear compared to those obtained via MRI. Moreover, due to the size limitations of the probe, we cannot examine patients with overly large breast or overlarge tumor. Moreover, we cannot eliminate the interference caused by the shadow effect to determine tumor boundaries in lumps behind the nipple and lumps with low echo signals or significant rear signal attenuation, resulting in measurement errors. In a few cases, manual two-dimensional tumor sectioning should have been added during ABVS dynamic continuous scanning to facilitate the manual segmentation of the 3D reconstruction and to supplement the missing images of the tumor boundary on the vertical section of the probe. The direction change of the tumor induced by gravity was measured using the 3D reconstruction. Statistical analysis showed that compared with the horizontal and coronal positions, the mean value of MRI measurements was significantly greater than that of US measurements in the sagittal position. This finding was consistent with the changes in the direction of gravity in the two patient positions. We analyzed the changes in the positional relationship between the tumor and nipple in both positions of the patients and emphasized the importance of patient positioning in the breast examination.

Our study has several limitations. First, as mentioned before, our method has certain restrictions in terms of breast and tumor size. Patients with overly large breast or tumors cannot be evaluated. Second, pathological results were provided by a pathologist who followed our measurement requirements and we could not obtain the 3D structure of the histopathology due to lack of corresponding values on the 3D warp knits. Therefore, we could not evaluate the degree of conformity between tumor morphology and pathological specimens because the histopathological results could not be compared with the tumors reconstructed via MRI and US. Third, we used localized solid space-occupying lesions with clear boundaries in the present study and did not include cases with diffuse lesions and unclear boundaries. This made our cohort relatively homogeneous and did not compare breast neoplasms of different morphologies and pathological types. Fourth, the sample size was small.

In conclusion, we innovatively designed a novel method that allowed us to obtain a full 3D US reconstruction of the unilateral breast structure. However, the information we provided is descriptive rather than conclusive. Therefore, further research is warranted to confirm these findings.

While we were aware of these limitations, we also see the reliability of our new method for ultrasound three-dimensional reconstruction of the breast as it accurately presents the tumor size in a pathologically controlled study. We have presented a novel US 3D reconstruction method for evaluating tumor size, which can provide a basis for investigated advanced visualization techniques for assessing breast tissue such as holographic presentation of 3D image data. These methods can provide physicians with a novel approach for making accurate surgical plans, for better communication with patients and for more effective navigation throughout the operation.
